# Body Composition in Inflammatory Bowel Disease

**DOI:** 10.34172/aim.2023.26

**Published:** 2023-03-01

**Authors:** Hongyun Wei, Ziying Yuan, Keyu Ren, Yanchun Jin, Linlin Ren, Bin Cao, Yuanyuan Zhou, Linlin Chen

**Affiliations:** ^1^Department of Gastroenterology, Affiliated Hospital of Qingdao University, Qingdao, Shandong 266000, P.R. China; ^2^The Fourth Department of the Digestive Disease Center, Suining Central Hospital, Suining, Sichuan 629000, P.R. China

**Keywords:** Body composition, Clinical outcomes, Inflammatory bowel disease, Treatment strategies

## Abstract

Inflammatory bowel disease (IBD) is associated with body composition changes, which are associated with clinical prognosis, response to therapy, and quality of life in IBD patients. Therefore, it is critical to review the body composition distribution in IBD, summarize the potential factors affecting body composition distribution, and take steps to improve the body composition distribution of IBD patients as early as possible. In the current review, we searched PubMed via keywords ‘inflammatory bowel disease’, or ‘IBD’, or ‘Crohn’s disease’, or ‘CD’, or ‘ulcerative colitis’, or ‘UC’, and ‘body composition’. Malnutrition and sarcopenia are common in IBD patients and are associated with the clinical course, prognosis, and need for surgery. Disease activity, reduced nutrition intake, vitamin D deficiency, and intestinal dysbiosis are factors contributing to changed body composition. Early use of biological agents to induce remission is critical to improving body composition distribution in IBD patients, supplementation of vitamin D is also important, and moderate physical activity is recommended in IBD patients with clinical remission.

## Introduction

 Inflammatory bowel disease (IBD) is a non-specific chronic relapsing bowel inflammatory disease, including ulcerative colitis (UC) and Crohn’s disease (CD). UC affects the colon, whereas CD can involve any component of the gastrointestinal tract from the mouth to the perianal area. It has long been identified that patients with IBD have altered body composition during various disease states. It is important to evaluate body composition in IBD patients precisely.

 Body mass index (BMI) and fat-free mass index (FFMI) are the common indices used for evaluating malnutrition. Low BMI or FFMI were commonly found in IBD patients despite being well-nourished,^1^ and were reported to be associated with worse prognosis, longer hospital stay, and decreased quality of life in IBD patients.^2^ About 37% of CD patients and 20% of UC patients have low BMI; low FFMI occurred in 28% of CD and 13% of UC patients.^3^ A study including 54 CD patients and 87 UC patients showed that most of the patients were eutrophic and overweight using BMI,^4^ but rates of myopenia and osteopenia were high.^5^ BMI is unable to distinguish between fat mass and fat-free mass. In addition, visceral fat area or index, not BMI, was negatively associated with infliximab trough concentration in patients with CD.^6^ The mean BMI of patients with CD in a cross-sectional study was lower than healthy control subjects, while visceral fat/BMI ratio was significantly higher in the CD group than controls.^7^ Furthermore, a recent study showed the male patients of the participating CD had significantly higher BMI compared to the general healthy Irish population, while lean tissue mass was similar.^8^ In addition, the majority of body fat was located in the abdominal or trunk region,^8^ such as mesenteric creeping fat. All the above studies indicate that BMI cannot account for fat distribution without distinction between lean and fat mass. Therefore, it is critical to study body composition distribution in IBD patients. We searched PubMed with the keywords ‘body composition’ and ‘inflammatory bowel disease’ or ‘Crohn’s disease’ or “ulcerative colitis”, or ‘CD’, or ‘UC’, and aimed to review the distribution and role of body composition in IBD, and tried to summarize potential strategies for improving body composition in IBD patients.

## Body Composition Distribution in IBD

 IBD patients had a significant malnutrition risk compared to healthy children and adolescents after matching for gender and age via phase angle assessment.^9^ Diet intake was associated with nutritional status in IBD patients. Most IBD patients changed their diet to avoid digestive symptoms, and 46% of these IBD participants had a nutritional deficiency, including iron or calcium deficiency.^10^ Most IBD patients had malnutrition or were myopenic,^11^ both in adults or children.

 In one study, 58% of patients with CD had sarcopenia, 21.6% had malnutrition, and 19.3% had visceral obesity based on computed tomography at the third lumbar vertebra level.^12^ A study showed that 24% of young adults with childhood-onset IBD had myopenia, and 9% were myopenic-obese; these profiles were strongly associated with low bone mineral density (BMD).^13^

 According to another study, 21.4% of 173 IBD patients were at risk of malnutrition according to the malnutrition universal screening tool, and 27.7% had sarcopenia.^14^ Interestingly, patients with CD had lower FFMI values compared to UC patients, especially male patients or those with small bowel involvement CD.^14^ Low BMI values were not gender-specific, but substantially more female CD patients had low FFMI than male CD patients.^15^ Furthermore, the study showed sarcopenia to be significantly associated with more hospitalizations, digestive surgery, and abscesses compared to non-sarcopenic patients.^12^ In summary, malnutrition and sarcopenia are common in IBD patients and might be associated with clinical outcomes.

## Relationship Between Body Composition and Prognosis of IBD

 Patients with IBD had significantly reduced skeletal muscle mass and a higher percentage of asthenia with decreased life quality.^16^ The decline of FFMI was significantly associated with IBD disease duration.^17^ Furthermore, phase angle was positively correlated with disease activity in patients with CD.^18^ Also, a higher visceral fat index increased the risk of stricturing/penetrating phenotypes in patients with CD.^19,20^ The combination of sarcopenia and high visceral fat could predict worse outcomes in patients with CD than either.^21^ Lean mass and muscle strength, but not fat mass, significantly correlated with regional and whole body BMD, but lean mass was the only independent predictor of hip BMD after multiple regression analysis.^22^ Lean mass was found to be an independent predictor of BMD at the hip whereas appendicular muscle mass (AMM) was independently predictive of the hip, forearm, and total body BMD.^22^

 Malnutrition was likely to occur in CD patients at the time of diagnosis, especially children, and lean body mass would increase after treatment.^23^ In addition, a diagnosis of childhood-onset IBD was an independent factor associated with low BMD and body composition.^24^

 In a recent study, low albumin levels and psoas muscle index of 187 IBD patients were related to prolonged hospitalization. In 99 CD patients on admission, a high ratio of visceral-to-subcutaneous adipose tissue area and low psoas muscle index were associated with intestinal resection, especially in male CD patients.^25^ Another study showed that 56.1% of UC in patients with sarcopenia required medical rescue and/or colectomy compared to 28% of patients without sarcopenia, indicating that sarcopenia could act as a predictor of need for rescue therapy.^26^

 Furthermore, sarcopenia was a predictor of need for surgery in IBD patients with BMI ≥ 25 based on univariate analysis, and the association between surgery and sarcopenia was significant (*P* = 0.002), while C-reactive protein was a significant predictor of need for surgery in normal or underweight patients according to multivariable analysis.^27^ In addition, sarcopenia also predicted surgery and postoperative complications in IBD patients.^28^ In addition, a high visceral-to-subcutaneous fat ratio was related to postoperative infectious complications of IBD patients.^29^ Sarcopenic UC patients were more likely to need to switch to colectomy than patients without malnutrition, acting as an independent risk factor for predicting colectomy.^30^ Therefore, improving the nutritional status of IBD patients could help induce disease remission.

## Potential Factors Affecting Body Composition in IBD

 It is necessary to clarify the possible factors affecting the distribution of body composition, which will help choose appropriate treatment strategies to improve body composition and prognosis.

 According to a recent study, most patients with IBD avoided some foods to prevent disease relapse. Therefore, malnutrition patients had decreased muscular strength than well-nourished ones.^31^ In addition, patients with IBD have reduced intestinal absorption, especially in CD patients with small intestine involvement or extensive bowel resection.

 Intestinal dysbiosis, especially small intestinal bacterial overgrowth, is common in IBD patients.^32^ Intestinal dysbiosis-associated inflammation could lead to malabsorption.^33^

 Creeping fat is a typical feature of CD,^34^ and is associated with disease severity of CD through activation of the immune response.^35^ Inflammatory cytokines could lead to protein degradation in muscles via decreasing the expression of insulin-like growth factor 1 and resistance to growth hormones of muscles, such as tumor necrosis factor α (TNF-α) and Interlukin-6.^36^ Sixty percent of IBD patients were reported to have vitamin D deficiency, especially in CD patients,^37,38^ and pediatric CD patients with vitamin D deficiency are prone to suffering from sarcopenia.^39^

## Potential Strategies for Improving Body Composition in IBD Patients

 The prevalence of sarcopenia was higher in UC patients with high Mayo scores, and medical treatment or surgery could reverse sarcopenia.^40^

 Infliximab reverses inflammatory muscle wasting (sarcopenia) in CD,^41^ indicating that infliximab should be used earlier in children CD. Infliximab, as an anti-TNFα agent, could increase muscle volume and strength in the legs after 25 weeks.^41^ CD patients on infliximab therapy showed a higher fat-free mass compared to traditional therapy,^42^ indicating that the use of biological agents could improve the body composition distribution. The presence of myopia was associated with primary non-response to anti-TNF-α therapy, which might offer an alternative dosing paradigm.^43^

 In addition, resting energy expenditure increased in stable CD patients compared with healthy control when adjusted for free fat mass. Furthermore, immunosuppressive therapy could reduce resting energy expenditure by regulating inflammation. Also, enteral nutrition could significantly improve the body composition, and nutritional status after 52 weeks.^44^

 Additionally, moderate-intensity combined aerobic and resistance training could improve body composition compared to the non-exercising group in IBD patients with clinical remission.^45^

 In pediatric CD patients, leg lean mass and muscle strength were associated with ongoing disease activity, and greater time of moderate to vigorous physical activity could improve lean mass.^46^ Therefore, early treatment to induce remission and moderate physical activity in IBD patients is recommended.

 In conclusion,malnutrition and sarcopenia are common in IBD patients, especially in pediatric IBD patients. Malnutrition is associated with longer hospitalization, disease activity, and the need for surgery. Early use of biological agents to induce remission is critical to improving body composition distribution in IBD patients, supplementation of vitamin D is also important, and moderate physical activity is recommended in IBD patients with clinical remission.
